# Editorial: Why not a history?

**DOI:** 10.1120/jacmp.v11i1.3261

**Published:** 2010-01-28

Medical physics is now a mature profession. A few years ago, my country's scientific medical physics organization, the American Association of Physicists in Medicine (AAPM), celebrated its 50th anniversary with all the pomp and hoopla befitting a golden anniversary. Medical physics as a recognized profession is even older than 50 years, having predated the formation of the AAPM by perhaps 20 to 30 years. Regardless of the specific date of the origins of our profession, medical physics is no longer a young profession, but one that has a history. The time has come to formally put this history down on paper.

Such a document does not yet exist. Some articles have appeared in journals retelling stories about medical physicists and their practice, and the AAPM has generated a videotaped set of interviews with key senior individuals in our profession. However, these have all been conducted by medical physicists, in general, those who have been directly involved as participants in the making of these histories. None of these accounts have been written by professional historians who can view the history of medical physics from an unbiased point of view. Moreover, all of these histories are of the natures of “names, dates, and places,” and none show the sort of analysis one would expect from a professional in the field.

In my opinion, a thorough history of medical physics needs to address several issues, none of which is likely to be addressed in an unbiased manner from within the fold of medical physicists. These issues require the services of a professional historian in order to be addressed adequately. One such issue is the continual conflict within our profession to answer the question, “Are medical physicists primarily scientists in medicine or primarily health care professionals whose background is in physics?” – a question that has been the origin of many debates in our profession. For example, the ongoing debate in the US regarding professional training of medical physicists is a direct consequence of this question. Those who advocate the development of a professional doctorate degree fall on one side of the question, whereas those who oppose the degree fall on the other side. Another example of this conflict is the debate regarding provider status for medical physicists. The ability of medical physicists to bill patients directly for medical physics services is a clear reflection of medical physicists as health care professionals. Many more controversies in our profession can be traced directly to this question of primary identification, and could be addressed in an unbiased history of medical physics.

Another issue that should be addressed in a history of our profession is the determination of professional status for medical physicists. What has our relationship been with our physician colleagues? Or have they really been our colleagues? Are we viewed by physicians as glorified and highly‐paid technicians, or are we viewed as equals in the delivery of health care, or are we somewhere in between? Are medical physicists viewed differently in different countries, and how has this viewpoint changed with time, if at all? What about turf battles with other health care professionals such as dosimetrists and physics assistants?

I would like to see an appropriate medical physics organization (AAPM, IOMP, or the like) set aside some funds to commission a professional historian to write a definitive history of our profession. We can continue the fine work that is being done by medical physicists who are documenting our history, but we need to realize that we are amateurs at this. Let us recognize that a professional historian who is addressing medical physics as an unbiased observer can bring a great deal of insight to the project and help us achieve a better understanding of who we are and where we are heading as a profession.

I shall be looking forward to the book.

George Starkschall, PhD, Editor‐in‐Chief

February 15, 2010

